# Real-World Metastatic Renal Cell Carcinoma Treatment Patterns and Clinical Outcomes in The Netherlands

**DOI:** 10.3389/fphar.2022.803935

**Published:** 2022-03-23

**Authors:** S.A. van Laar, K.B. Gombert-Handoko, R.H.H. Groenwold, T. van der Hulle, L.E. Visser, D. Houtsma, H.J. Guchelaar, J. Zwaveling

**Affiliations:** ^1^ Department of Clinical Pharmacy and Toxicology, Leiden University Medical Center, Leiden, Netherlands; ^2^ Department of Clinical Epidemiology, Leiden University Medical Center, Leiden, Netherlands; ^3^ Department of Medical Oncology, Leiden University Medical Center, Leiden, Netherlands; ^4^ Department of Hospital Pharmacy, Haga Teaching Hospital, The Hague, Netherlands; ^5^ Department of Epidemiology, Erasmus MC, Rotterdam, Netherlands; ^6^ Department of Hospital Pharmacy, Erasmus MC, Rotterdam, Netherlands; ^7^ Department of Internal Medicine, Haga Teaching Hospital, The Hague, Netherlands

**Keywords:** immune check inhibitor (ICI), renal cell carcinoma (RCC), tyrosine kinase inhibitor (TKI), text-mining, electronic health record (EHR)

## Abstract

The number of treatment options for patients with metastatic renal cell carcinoma (mRCC) has significantly grown in the last 15 years. Although randomized controlled trials are fundamental in investigating mRCC treatment efficacy, their external validity can be limited. Therefore, the efficacy of the different treatment options should also be evaluated in clinical practice. We performed a chart review of electronic health records using text mining software to study the current treatment patterns and outcomes. mRCC patients from two large hospitals in the Netherlands, starting treatment between January 2015 and May 2020, were included. Data were collected from electronic health records using a validated text mining tool. Primary endpoints were progression-free survival (PFS) and overall survival (OS). Statistical analyses were performed using the Kaplan–Meier method. Most frequent first-line treatments were pazopanib (n = 70), sunitinib (n = 34), and nivolumab with ipilimumab (n = 28). The overall median PFS values for first-line treatment were 15.7 months (95% confidence interval [95%CI], 8.8–20.7), 16.3 months (95%CI, 9.3–not estimable [NE]) for pazopanib, and 6.9 months (95% CI, 4.4–NE) for sunitinib. The overall median OS values were 33.4 months (95%CI, 28.1–50.9 months), 39.3 months (95%CI, 29.5–NE) for pazopanib, and 28.1 months (95%CI, 7.0–NE) for sunitinib. For nivolumab with ipilimumab, median PFS and median OS were not reached. Of the patients who finished first- and second-line treatments, 64 and 62% received follow-up treatments, respectively. With most patients starting on pazopanib and sunitinib, these real-world treatment outcomes were most likely better than in pivotal trials, which may be due to extensive follow-up treatments.

## 1 Introduction

**FIGURE 1:**
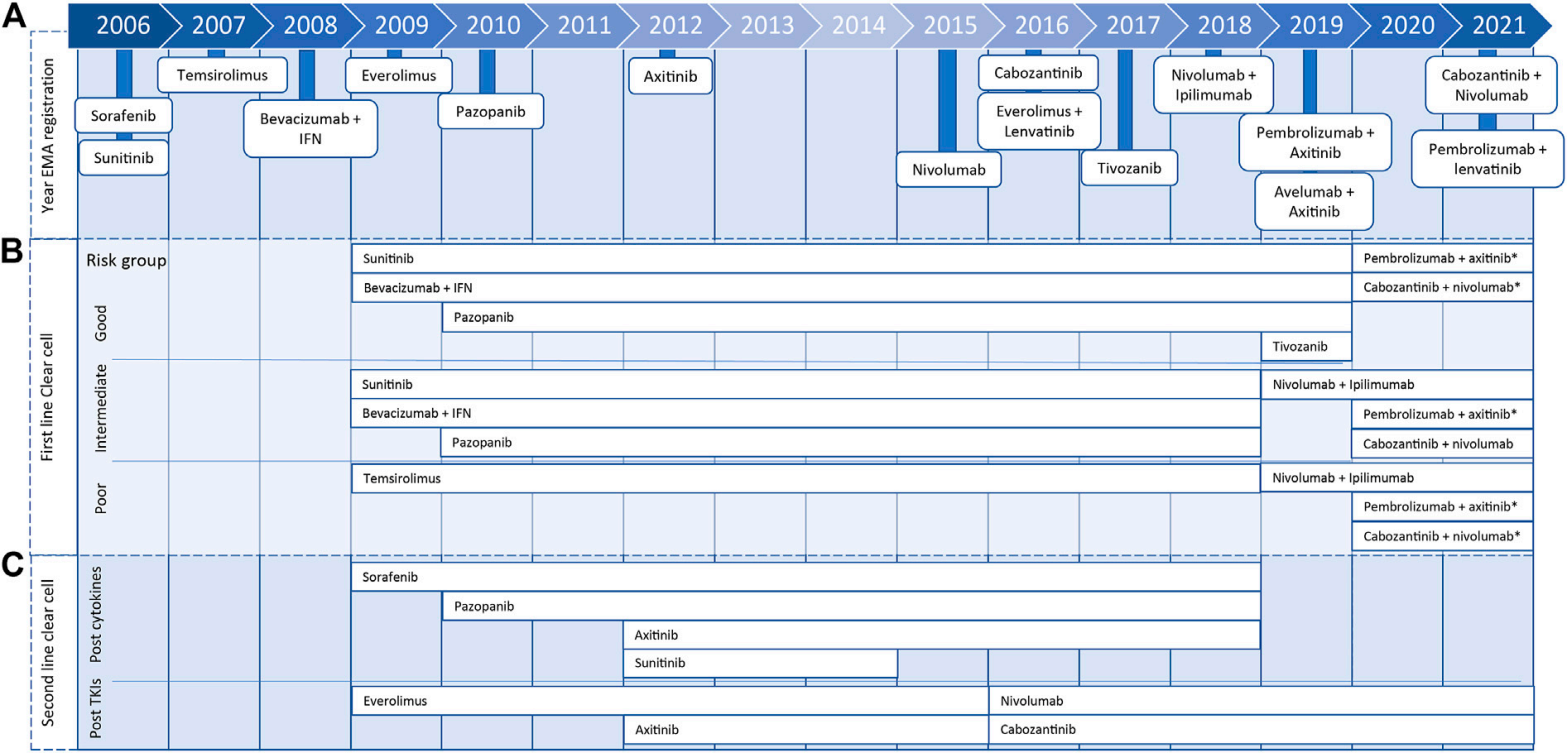
Timeline presenting year of marketing authorization by European Medicines Agency of metastatic renal cell carcinoma treatments **(A)** and years in which treatment was included for clear cell renal cell carcinoma treatment in ESMO Clinical Practice Guidelines for diagnosis, treatment, and follow-up for first **(B)** or second line **(C)**, including 2020 eUpdate (*).

Yearly, more than 400,000 patients worldwide are diagnosed with kidney cancer, of which 90% of the tumors are classified as renal cell carcinoma (Janzen et al., 2003; Ferlay et al., 2019). Metastatic renal cell carcinoma (mRCC) is not susceptible to chemotherapy or hormonal therapy, and until the introduction of the targeted therapy, the first-line treatment of mRCC was cytokine therapy with interleukin-2 or interferon-alpha (Oudard et al., 2007; Hutson, 2011). Sunitinib was the first targeted therapy that obtained marketing authorization for the treatment of mRCC by the European Medicines Agency in 2006. Subsequently, 14 other targeted therapies, tyrosine kinase inhibitors (TKIs), mammalian target of rapamycin inhibitors, and immune checkpoint inhibitors, were approved. Recently, five new combination treatments were registered for first-line mRCC patients (Figure 1). Patients with mRCC have a median overall survival (mOS) between 22.9 and 29.1 months on TKIs. For immunotherapy (nivolumab with ipilimumab), only in the intermediate/poor risk group, mOS is reached and is 48.1 months (Choueiri and Motzer, 2017; Albiges et al., 2020; Nocera et al., 2022).

In 2008, the first European Society for Medical Oncology Clinical Practice Guideline for diagnosis, treatment, and follow-up of RCC was published and has been updated almost yearly (Escudier et al., 2019). From 2008 until 2017, the standard of care for patients with clear cell mRCC with a good or moderate prognosis score, according to the Memorial Sloan Kettering Cancer Center or International mRCC Database Consortium (IMDC) risk model, was first-line treatment with TKIs or bevacizumab with interferon. For patients with a poor prognosis score, the standard was treatment with temsirolimus. In 2019, the advised first-line treatment for patients with an intermediate and poor prognosis score changed to nivolumab with ipilimumab. Subsequently, the recommendation for patients with a good prognosis changed in 2020 to pembrolizumab with axitinib or cabozantinib with nivolumab. Both treatment combinations were also added to the intermediate and poor prognosis groups (Figure 1). Furthermore, nivolumab and cabozantinib were introduced as second-line in clear cell mRCC treatment options (Figure 1) (Escudier et al., 2019; ESMO Guidelines Committee, 2020b).

Randomized controlled trials (RCTs) are the cornerstone for investigating the efficacy of cancer treatments and, therefore, the pivotal studies for marketing authorization application of mRCC treatments (Bothwell and Podolsky, 2016; Franklin and Schneeweiss, 2017; Verweij et al., 2019). However, due to the structured protocols, ideal setting, and inclusion of only a part of the target population, these studies may have limited external validity. Real-world evidence, based on real-world data from registries, databases, or observational studies, could be used to close the inferential gap between the RCTs on mRCC treatments and the clinical practice (Stewart et al., 2007; de Lusignan et al., 2015; Skovlund et al., 2018; Liu et al., 2019).

Electronic health records (EHRs) contain extensive medical information about patients in clinical practice and therefore are a potentially important resource to assess treatment effectiveness. However, the regular method for EHR data collection, manual chart review, is very time-consuming (Haerian et al., 2012; Casey et al., 2016; Assale et al., 2019). To improve efficiency in real-world data collection in the mRCC population, we investigated and validated a natural language processing and text mining software tool as a more efficient collection method in a cohort of mRCC patients receiving systemic drug treatment (Kibbelaar et al., 2017).

In this study, we applied this text-mining tool to investigate how the introduction of new treatment options for mRCC changed the clinical practice by evaluating treatment strategies and the efficacy of the different first-line treatment options.

## 2 Methods

### 2.1 Study Design

A retrospective cohort study was performed to assess the mRCC treatment patterns and treatment outcomes in clinical practice. Patients were included in the Leiden University Medical Center (LUMC), Leiden, and the Haga Hospital, The Hague, the Netherlands.

The study protocol was reviewed and approved by the Medical Ethics Review Committee of the LUMC, Leiden, who waived the need for informed consent.

### 2.2 Patients and Endpoints

All patients aged 18 years or older starting first-line systemic treatment for mRCC between January 2015 and May 2020 (LUMC) or January 2017 and May 2020 (Haga Teaching Hospital) were included. Primary endpoints were first-line progression-free survival (PFS) and overall survival (OS). OS was defined as the time from treatment initiation until death from any cause; patients were censored when alive at the moment of data collection. PFS was defined as the time from treatment initiation until the date of progression according to the reported PFS by the oncologist, which is in line with RECIST 1.1 (Eisenhauer et al., 2009) or death from any cause; patients were censored when still on treatment at the moment of data collection or when treatment ended for other reasons.

The following patient characteristics were collected at the start of treatment: age, sex, performance status (PS) [Karnofsky—or Eastern Cooperative Oncology Group PS], tumor histology, and prior nephrectomy. Additionally, the IMDC prognostic factors were collected; these include time from diagnosis to systemic therapy within 1 year, Karnofsky PS below 80%, hemoglobin level below the lower limit of normal and corrected calcium levels, neutrophil levels, and platelet levels above the upper limit of normal (Heng et al., 2009; Heng et al., 2013). All systemic mRCC drug treatments, with a concomitant date of progression and date of death as treatment outcome parameters, were collected.

### 2.3 Data Retrieval

Patient inclusion and data collection were performed using a natural language processing and text mining software tool Clinical Data Collector (CTcue B.V., Amsterdam, the Netherlands). Both the details of the software and the validation process were described previously (van Laar et al., 2020). In short, for patient selection, characteristics, and outcomes, queries were constructed and validated by comparison to manual review.

### 2.4 Statistical Analysis

Statistical analysis was performed using R (R, 2019, Vienna, Austria). Descriptive statistics were used to describe the total patient cohort and subgroups of first-line treatments with more than 10 patients. Multiple imputations from SPSS (IBM SPSS Statistics for Windows, version 25.0. Armonk, NY: IBM Corp.) was used to correct for missing data on PS and neutrophil count. Subgroup distribution of variables was compared using the chi-square and analysis of variance tests. Additionally, for the visualization of the treatment patterns, a Sankey plot was created. OS and PFS from first-line treatment were analyzed using the Kaplan–Meier method and summarized by medians, 95% confidence intervals (CIs), and Kaplan–Meier survival plots. All statistical analyses were exploratory.

## 3 Results

In total, 138 patients receiving first-line systemic treatment for mRCC were included in this observational study. Overall, the patient population had a median age of 67 years and was 75% men. Half of the patients had a prior nephrectomy, and in 73% of patients, clear cell histology was found. According to the IMDC criteria, 15% of patients had a good prognosis, 41% of patients had an intermediate prognosis, and 44% had a poor prognosis (Table 1).

**TABLE 1:** Patient characteristics of complete study population and first-line sunitinib, pazopanib, and nivolumab with ipilimumab.

	Total population n = 138, *n (%)*	First-line sunitinib n = 34 (25%), *n (%)*	First-line pazopanib n = 70 (51%), *n (%)*	First-line nivolumab with ipilimumab n = 28 (20%), *n (%)*	*p*-Value
Sex, male	103 (75)	24 (71)	58 (83)	17 (61)	0.058
Age, median (1st and 3rd quarter)	67 (59–73.75)	64 (58–72.5)	71 (64–76)	63 (57–66.5)	<0.01 *
Previous nephrectomy	71 (51)	15 (44)	47 (67)	7 (25)	<0.01*
Histological subtype of renal cell carcinoma					0.034*
Clear cell	101 (73)	19 (56)	56 (80)	23 (82)	
Other					
*Papillary*	*6 (4)*	*3 (9)*	*3(4)*	*0*	
*Sarcomatoid*	*5 (4)*	*3 (9)*	*0*	*0*	
*Chromofobic carcinoma*	*1 (1)*	*0*	*0*	*0*	
*Missing*	*25 (18)*	*9 (27)*	*11 (16)*	*5 (18)*	
IMDC risk score parameters					
Hypercalcemia	54 (38)	10 (29)	24 (34)	16 (57)	0.054
Anemia	88 (64)	25 (74)	40 (57)	17 (61)	0.267
Missing/imputed	3 (2)	1 (3)	0	0	
Neutrophilia	36 (26)	12 (35)	19 (27)	8 (29)	0.69
Thrombocytosis	30 (22)	10 (29)	11 (16)	8 (29)	0.18
Performance status <80% Karnofsky	15 (11)	11 (32)	16 (23)	5 (18)	0.38
Missing/imputed	57 (41)	15 (44)	38 (54)	3 (11)	
Time from diagnosis to systemic therapy <1 year	72 (52)	20 (59)	27 (39)	21 (75)	<0.01 *
IMDC risk group					0.175
Favorable risk (0 points)	20 (15)	3 (9)	15 (21)	2 (7)	
Intermediate-risk (1–2 points)	57 (41)	13 (38)	30 (43)	11 (39)	
Poor-risk (>2 points)	61 (44)	18 (53)	25 (36)	15 (54)	

Abbreviations: IMDC, International Metastatic renal cell carcinoma Database Consortium; *p = < 0.05.

### 3.1 Treatment Patterns

**FIGURE 2:**
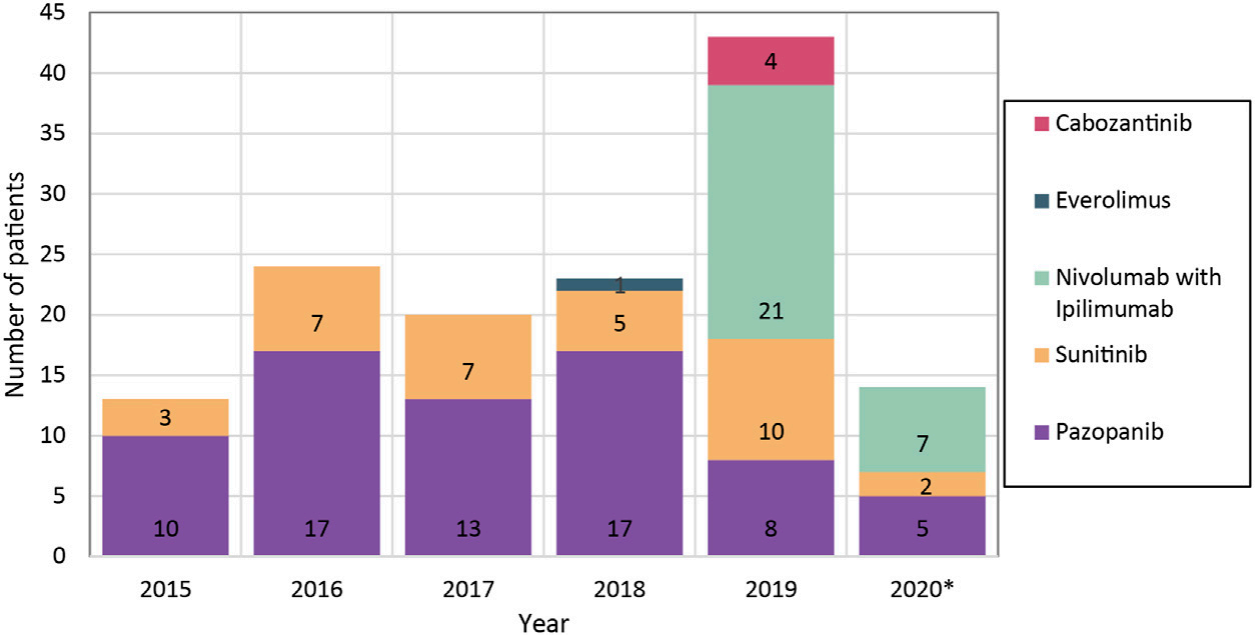
Number of first-line treatments started per year. *Patients included until May 2020.

From 2015 until 2018, 13 to 24 patients per year started a first-line treatment (Figure 2). In this period, approximately 80% of the patients received pazopanib as first-line treatment; the other patients mainly received sunitinib. In 2019, the patients starting with a first-line treatment raised to 43, and approximately half of these patients underwent first-line treatment with nivolumab and ipilimumab.

**FIGURE 3:**
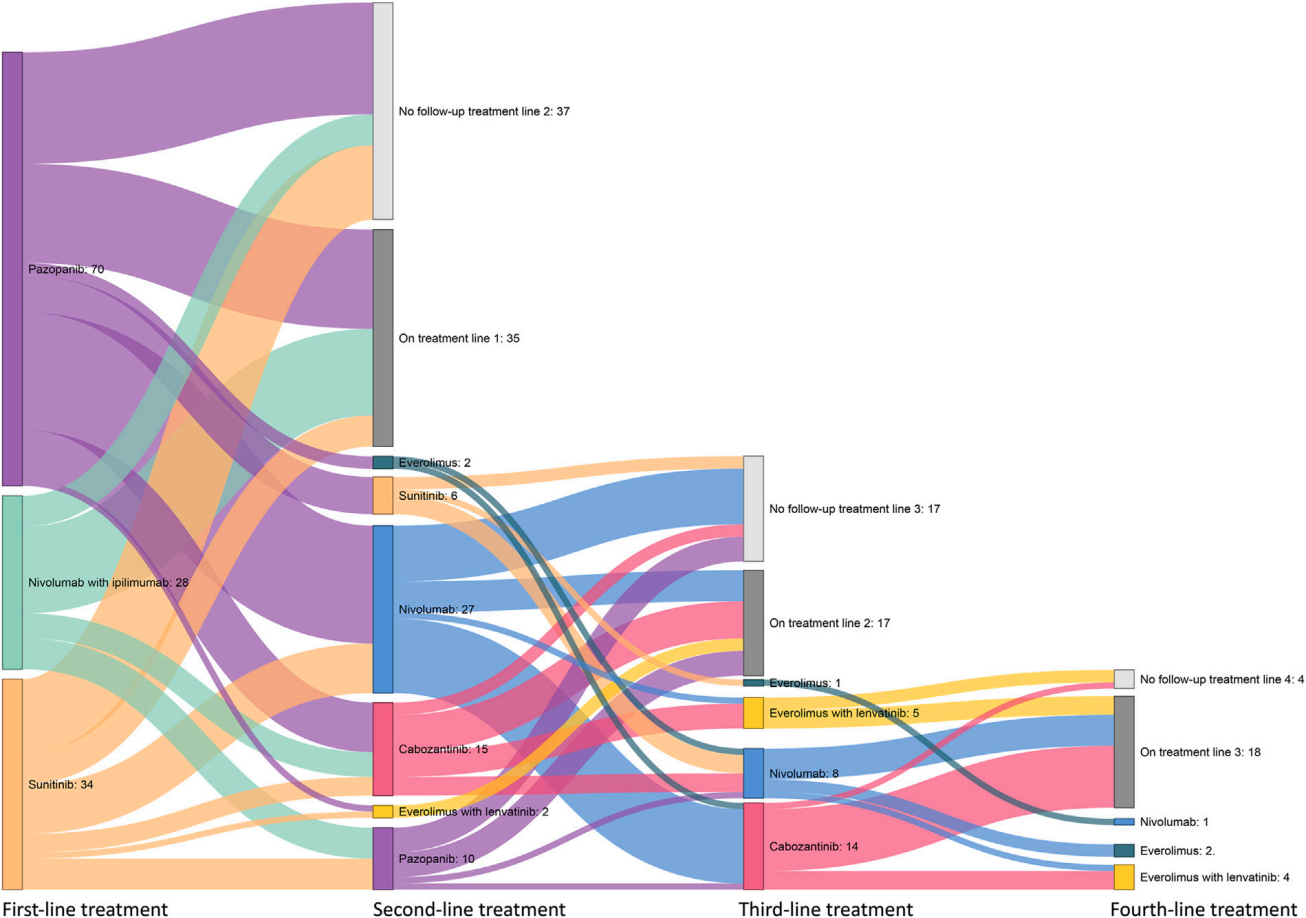
Overview of systemic drug treatment patterns following patients who received first-line pazopanib, sunitinib, or nivolumab with ipilimumab treatment.

Overall, most patients received pazopanib (n = 70, 51%), sunitinib (n = 34, 24%), or nivolumab with ipilimumab (n = 28, 20%) as first-line treatment. Figure 3 shows the treatment patterns of the patients who started on nivolumab with ipilimumab, pazopanib, and sunitinib. At the moment of data collection, 35 patients were still on first-line treatment and 17 patients and 18 on second- and third-line treatments, respectively. In 62 patients (64% of the patients who ended first-line treatment) who received a second-line treatment, nivolumab (n = 27, 44%), cabozantinib (n = 15, 24%), and pazopanib (n = 10, 16%) were mostly used. In the third-line treatment, 14 of the 28 patients (62% of the patients who ended second-line treatment) received cabozantinib. Of the 11 patients who ended third-line treatment, eight (73%) received four or more systemic treatments, in which everolimus with lenvatinib was the most used.

### 3.1 Treatment Outcomes

#### 3.1.1 Patients and Patient Characteristics

Subgroup comparison of patient characteristics at start of the first-line treatment on pazopanib, sunitinib, and nivolumab with ipilimumab showed significant differences in median age (P: 71 years, S: 64 years, N + I: 63 years; *p* < 0.01), previous nephrectomy (P: 67%, S: 44%, N + I: 25%; *p* < 0.01), known histological subtypes (P: 95% clear cell; 5% papillary, S: 76% clear cell; 12% papillary; 12% sarcomatoid, N + I 100% clear cell; *p* = 0.034), and time from diagnosis to systemic therapy within a year (P: 39%, S: 59%, N + I: 75%; *p* < 0.01).

#### 3.1.2 Progression-Free Survival and Overall Survival

**FIGURE 4:**
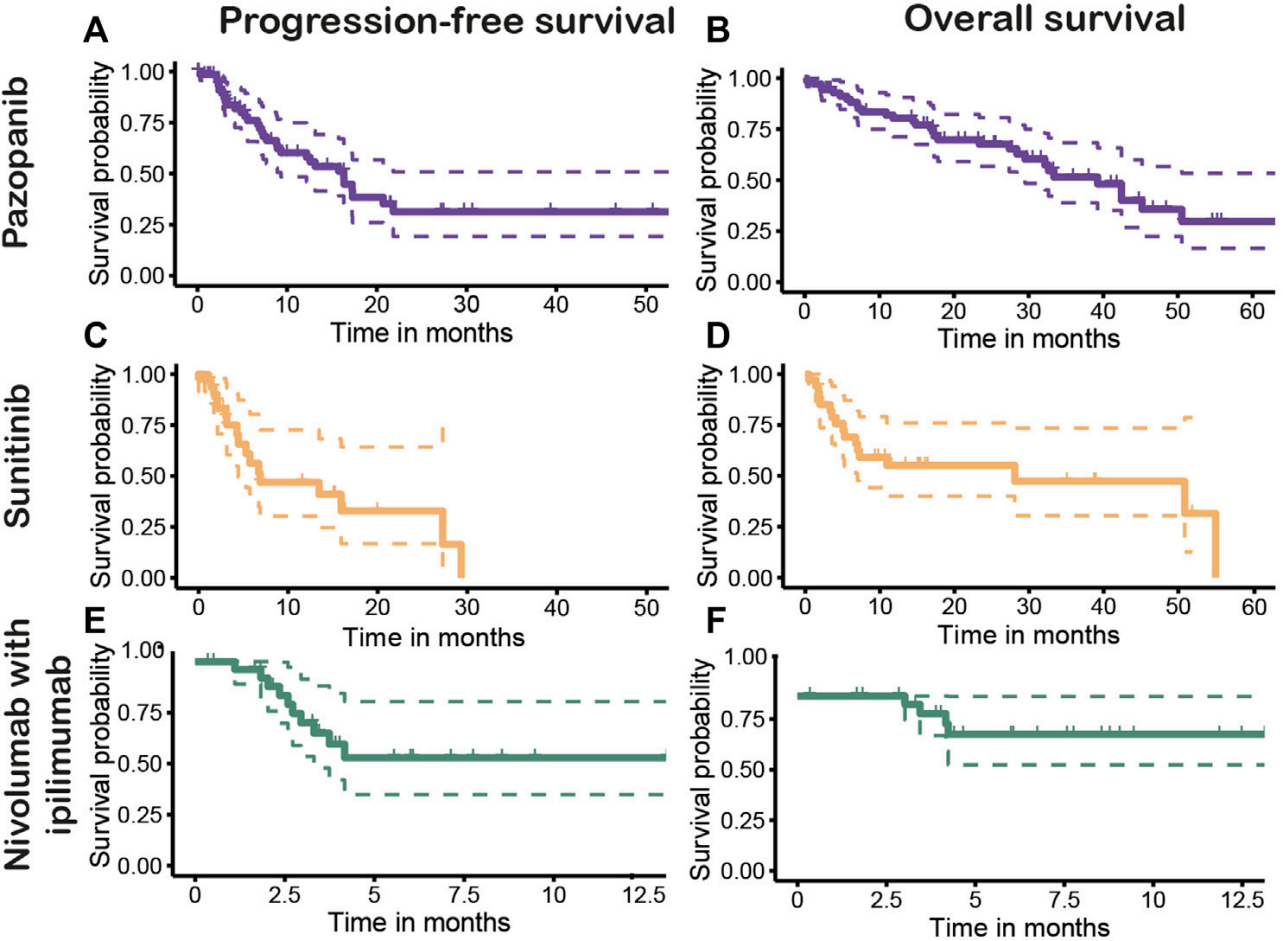
Progression-free (PFS) and overall survival (OS) of first-line treatments pazopanib [**(A)**: PFS, **(B)**: OS], sunitinib [**(C)**: PFS, **(D)**: OS], and nivolumab with ipilimumab [**(E)**: PFS, **(F)**: OS].

**FIGURE 5:**
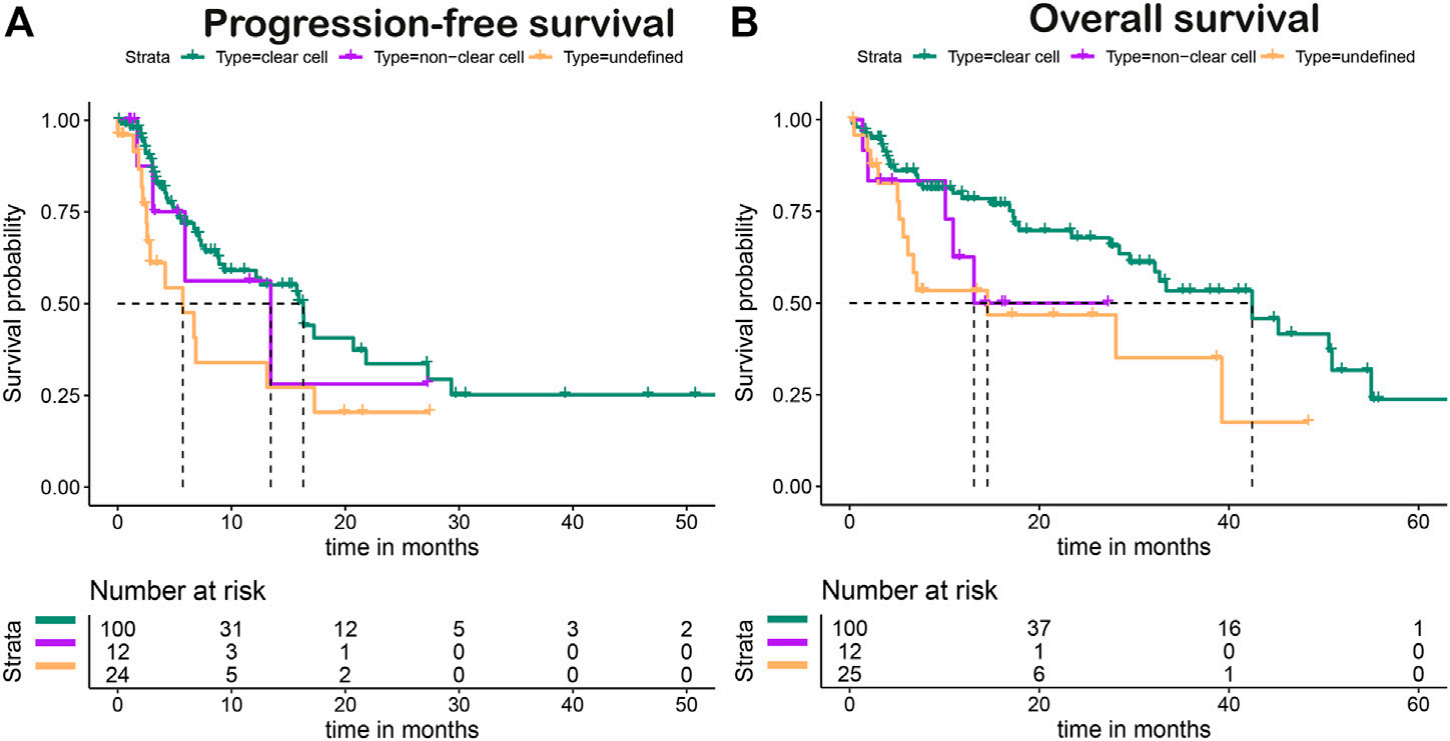
Progression-free **(A)** and overall **(B)** survival of clear cell *versus* non-clear cell histology.

Figure 4 shows the PFS and OS Kaplan–Meier plots stratified for first-line treatment. The overall median PFS after first-line treatment was 15.7 months (95% CI, 8.8–20.7). Patients with first-line pazopanib had a median PFS (mPFS) of 16.3 months (95% CI, 9.3–not estimable [NE]) (Figure 4A), and for patients receiving sunitinib, an mPFS of 6.9 months (95% CI, 4.4–NE) was observed (Figure 4C). No mPFS could be estimated for the treatment of nivolumab with ipilimumab due to the limited follow-up time (Figure 4E). The overall median OS (mOS) for all patients was 33.4 months (95% CI, 28.1–50.9 months). The mOS values per treatment were 39.3 months (95% CI, 29.5—NE) for first-line treatment with pazopanib (Figure 4B) and 28.1 months (95% CI, 7.0—NE) after first-line sunitinib (Figure 4D). For nivolumab with ipilimumab, mOS could not be established, as the median is not yet reached (Figure 4F). The stratification to clear cell and non-clear histology showed mPFS values of 16.3 months (95% CI, 9.3–27.3) and 13.5 months (5.9–NE) (Figure 5A) and mOS values of 42.4 months (95% CI, 32.2—NE) and 13.1 months (95%CI, 10.9–NE) (Figure 5B), respectively.

## 4 Discussion

In this study, using an EHR text mining tool, we were able to evaluate treatment strategies and estimate treatment effectiveness in patients with mRCC in daily practice. After first-line treatment with pazopanib (n = 70) and sunitinib (n = 34), the mPFS values were 16.3 and 6.9 months, and mOS values were 39.3 and 28.1 months, respectively. For nivolumab with ipilimumab (n = 28), the mPFS and mOS could not be determined yet.

The pivotal trial of pazopanib by Sternberg et al. (2010, 2013) showed an mPFS of 11.1 months for the treatment-naïve population and an mOS of 22.9 months. Motzer et al. (2006, 2007) showed an mPFS of 11 months and mOS 26.4 months after first-line sunitinib treatment. Additionally, the COMPARZ-trial, comparing first-line pazopanib with sunitinib prospectively, showed an mPFS of 10.5 *vs*. 10.2 months and mOS of 28.4 *vs*. 29.3 months, demonstrating non-inferiority of pazopanib to sunitinib. (Motzer et al., 2013). All these RCTs only included patients with (predominant) clear cell histology. The overall mOS in our study (33.4 months) was considerably higher compared with those found in the RCTs, which may be attributed to a longer mOS in the pazopanib-treated patients. However, the mPFS of both treatments and the mOS after treatment with sunitinib seem to be comparable with those found in the pivotal trials.

Although sunitinib and pazopanib are both TKIs and often-used as first-line treatments, these subgroups should not be used for a head-to-head comparison, as the populations in our study differ on several patient characteristics (age, previous nephrectomies, tumor histology*,* and a number of patients who started systemic treatment within a year). The sunitinib population included more patients with worse scores for all characteristics, except age. Pazopanib was of preference in the higher age population, as the COMPARZ trial showed better safety quality-of-life profiles than sunitinib.

The patients in our real-world study differed from the RCTs concerning several patient characteristics. Patients treated with pazopanib, *e.g.*, had a higher median age (71 *vs*. 59 years) and fewer (partial) nephrectomies (67.1 *vs*. 89%), and more patients (27.3%) were assigned to an IMDC poor-risk group than in the pivotal study, which included only 3% of patients with a poor Memorial Sloan Kettering Cancer Center risk (Sternberg et al., 2010). For sunitinib-treated patients, we observed that, in contrast to the pivotal study, a significant part of our patients treated with sunitinib had non-clear cell histology. Furthermore, our population had fewer (partial) nephrectomies (44.1 *vs*. 91%), and at least 32% of the patients had a confirmed Karnofsky PS < 80% in contrast to 0% in the RCT (Motzer et al., 2006). Because all these differences in patient characteristics in the real world compared with RCTs are related to worse prognosis (Heng et al., 2013; Bhindi et al., 2018; Padala et al., 2020), they do not explain the promising effectiveness of sunitinib and pazopanib that we found in our real-world population in comparison with RCTs.

A longer mOS, but comparable mPFS, of pazopanib, as compared with the pivotal studies, is also seen in recent real-world studies (Kim et al., 2018; Schmidinger et al., 2019a, 2019b). The earlier systematic review and meta-analysis of Climent et al. (2018) showed that real-world pazopanib studies published until December 31, 2016, resulted in mPFS ranging from 8.1 to 15.9 months and mOS ranging from 16.1 to 31.0 months, with a pooled mPFS of 10.0 months and mOS of 22.7 months. Also, for sunitinib, an improved mOS is found in recent studies (Kim et al., 2018; Schmidinger et al., 2019b). However, our findings for sunitinib are more in accordance with the systematic review and meta-analysis of Moran et al. (2019), which showed a pooled mPFS of 9.4 months and mOS of 20.8 months for sunitinib with individual results for mPFS and mOS ranging from 7.5 to 11.0 and 6.8 to 33.2 months, respectively. Although mOS of sunitinib in our real-world situation is similar to the RCTs, this was unexpected given the patient characteristics, which are indicative of poor outcomes.

More and better follow-up treatments may be the most obvious explanation for the prolonged OS. In this study, we show that 64% of the patients who finish the first-line treatment receive a second line of treatment and congruent 62% a third line, among which the relative new treatments nivolumab and cabozantinib are the most used. Both cabozantinib and nivolumab have been shown to significantly improve mOS as second-line treatment, even after recurrent progression when compared with treatment with everolimus (Motzer et al., 2015; Choueiri et al., 2016). A real-world unselected patient group showed a median duration of cabozantinib treatment of 7.6 months (Albiges et al., 2021). In the pivotal trial for first-line pazopanib, only 30% of the patients received any post-progression systemic therapy, most often sunitinib and sorafenib (Sternberg et al., 2013), which is a considerably lower percentage of the patients than in the real-world situation. Additionally, Schmidinger et al. (2019b) showed that, evaluating real-world data of an Austrian population, 67.7% of the patients received second-line treatment and had improved OS when compared with the RCTs. They argue that an improved OS is related to the quality of the healthcare system in a country and reimbursement of subsequent treatments (Schmidinger et al., 2019b). For example, in Brazil and the United Kingdom, only 20 and 15.8% of the mRCC patients are treated with second-line systemic therapy, respectively, and in these countries, limited availability and costs are expected to be barriers to optimal treatment (Wagstaff et al., 2016; Bergerot et al., 2018).

Moreover, toxicity-related dose reduction was linked to better outcomes in the study of Wagstaff et al. (Escudier et al., 2017). Therefore, clinicians having more experience with treatments, such as sunitinib and pazopanib, may be more alert to apply dose reductions when necessary and, in this way, contribute to the overall better prognosis of these treatments in the real world with respect to earlier-stage RCTs.

Twenty-eight patients in our cohort started first-line treatment with nivolumab and ipilimumab in 2019. Because one of the hospitals was designated for nivolumab in combination with ipilimumab treatment, this resulted in an influx of new mRCC patients. In 92% of the patients of which the IMDC prognosis was known, the prognosis was intermediate or poor, according to the European Society for Medical Oncology guidelines. Therefore, this study shows that the expected paradigm shift from TKIs to immunotherapy in mRCC treatment has started in the group of patients with intermediate and poor IMDC prognosis (Calvo et al., 2019). However, no median survival data were reached in this study. Therefore, we were not able to verify the mPFS of 11.2 months and mOS of 48.1 months reached in the CheckMate-214 study in the intermediate and poor-risk patient groups (Albiges et al., 2020).

As far as we know, this is the first study in which a text mining tool is used for real-world data extraction from EHRs to study treatment patterns and outcomes of mRCC treatments. Using the text mining tool, this study could be performed more efficiently than by manual review (a mean of 12 *vs*. 86 min per patient). Also, most outcome measurements could be extracted with the desired high accuracy of at least 90% (van Laar et al., 2020). Because this method enables faster data extraction compared with a manual review, in the future, tools such as these could be repeatedly used to evaluate treatments. These data can illustrate who real-world patients are and how new treatments influence their survival. The data can be used on several levels (Venkatakrishnan et al., 2020): on the patient level, as additional information to share with a patient in the process of shared decision making (Bomhof-Roordink et al., 2019), on the hospital level, or, because these queries are transferrable to other hospitals, even multicenter level, for treatment evaluation and pursuit of value-based healthcare and patient-centered care (Tseng and Hicks, 2016).

This study has some limitations. First, although the EHR is an information-rich source containing longitudinal patient data, it is a secondary source (Casey et al., 2016). Therefore, the data quality is dependent on the healthcare professionals' documentation, and not all data desired for research may be documented. In this study, the PS was the least well documented and missing in 41.3% of the patients, which is often seen in chart reviews (Day et al., 2015; Noize et al., 2017). Also, patients in this study could have participated in other post-registration trials, *e.g.*, the DIET study (Lubberman et al., 2019). In addition, we were limited in the extraction of prognostic factors, *e.g.*, tumor load and location of metastases, as the EHR documentation was still too complex for the text mining tool.

### 4.1 Future Perspectives

This paper shows that the real-world population differs from the pivotal trial populations and that multiple factors may influence treatment outcomes. Using a text mining tool, a quick evaluation of the effectiveness of the treatments for mRCC carcinoma in real-world patients is possible. Therefore, repeated use of the queries from the EHR text mining tool can provide recurring information for physicians, useful for decision-making for the treatment of individual patients, now and in the future, in the field of mRCC carcinoma. This is especially relevant in the present field, as more treatment combinations are already there and are expected to come (ESMO Guidelines Committee, 2020a).

## 5 Conclusion

This study aimed to evaluate the treatment patterns and outcomes of mRCC patients. With most patients starting on treatment with pazopanib and sunitinib, the outcomes of these real-world patients most probably were better than expected from pivotal trials. The extensive follow-up treatments patients received may have contributed to the improved outcomes. The used EHR text mining method can be easily applied for evaluation of other treatments in clinical practice, which may be useful in the rapidly evolving field of renal cell carcinoma treatments.

## Data Availability

The raw data supporting the conclusion of this article will be made available by the authors without undue reservation.
